# Experimentally Constrained Mechanistic and Data-Driven Models for Simulating NMDA Receptor Dynamics

**DOI:** 10.3390/biomedicines13071674

**Published:** 2025-07-08

**Authors:** Duy-Tan J. Pham, Jean-Marie C. Bouteiller

**Affiliations:** 1 Center for Neural Engineering, Alfred E. Mann Department of Biomedical Engineering, Viterbi School of Engineering, University of Southern California, Los Angeles, CA 90007, USA; 2 Neural Systems Computational Modeling Lab, Alfred E. Mann Department of Biomedical Engineering, Viterbi School of Engineering, University of Southern California, Los Angeles, CA 90007, USA; 3 Institute for Technology and Medical Systems (ITEMS), Keck School of Medicine, University of Southern California, Los Angeles, CA 90007, USA

**Keywords:** NMDA-R, synapse, kinetic model, particle swarm optimization, look-up table

## Abstract

**Background**: The N-methyl-d-aspartate receptor (NMDA-R) is a glutamate ionotropic receptor in the brain that is crucial for synaptic plasticity, which underlies learning and memory formation. Dysfunction of NMDA receptors is implicated in various neurological diseases due to their roles in both normal cognition and excitotoxicity. However, their dynamics are challenging to capture accurately due to their high complexity and non-linear behavior. **Methods**: This article presents the elaboration and calibration of experimentally constrained computational models of GluN1/GluN2A NMDA-R dynamics: (1) a nine-state kinetic model optimized to replicate experimental data and (2) a computationally efficient look-up table model capable of replicating the dynamics of the nine-state kinetic model with a highly reduced footprint. Determination of the kinetic model’s parameter values was performed using the particle swarm optimization algorithm. The optimized kinetic model was then used to generate a rich input–output dataset to train the look-up table synapse model and estimate its coefficients. **Results**: Optimization produced a kinetic model capable of accurately reproducing experimentally found results such as frequency-dependent potentiation and the temporal response due to synaptic release of glutamate. Furthermore, the look-up table synapse model was able to closely mimic the dynamics of the optimized kinetic model. **Conclusions**: The results obtained with both models indicate that they constitute accurate alternatives for faithfully reproducing the dynamics of NMDA-Rs. High computational efficiency is also achieved with the use of the look-up table synapse model, making this implementation an ideal option for inclusion in large-scale neuronal models.

## 1. Introduction

Accurate synaptic models are essential in computational neuroscience to adequately capture the biological complexity that underlies learning. Among the various types of synapses, N-methyl-D-aspartate receptors (NMDA-Rs) hold particular significance, as they contribute to the mediation of excitatory neurotransmission, alongside AMPA receptors (AMPA-Rs), and play fundamental roles in synaptic plasticity, learning, and memory. NMDA-Rs are heterotetrameric ligand-gated ion channels most commonly expressed in a diheteromeric configuration of two obligatory glycine-binding GluN1 subunits and two glutamate-binding GluN2 subunits [1]. GluN2 subunits can be expressed as four different types (GluN2A–GluN2D), each with their own distinct gating properties [2]. For example, the potency of glutamate on the NMDA-R response is directly influenced by the expressed GluN2 subunit. The exponential decaying time constants of NMDA-R-mediated currents after a 1 ms application of 1 mM L-glutamate were measured to vary significantly with respect to the expressed GluN2 subunit: ~40–50 ms for GluN1/GluN2A, ~300–400 ms for GluN1/GluN2B and GluN1/GluN2C, and ~4 s for GluN1/GluN2D [1,3]. Furthermore, statistical models of these four different diheteromeric subunit compositions predict significantly different peak open probability amplitudes with relatively high values from GluN1/GluN2A and GluN1/GluN2B subunits and several orders of magnitude lower from GluN1/GluN2C and GluN1/GluN2D subunits [4]. Of these four diheteromeric subunits, the GluN1/GluN2A and GluN1/GluN2B configurations predominate in the adult brain, with synaptic sites typically expressing GluN2A subunits [5]. Therefore, we focused on modeling the GluN1/GluN2A subunit composition of NMDA-Rs.

Modeling NMDA-R dynamics accurately presents a difficult challenge, since NMDA-Rs exhibit complex non-linearity arising from voltage-dependent magnesium blocks, dependence on other molecular species (such as co-agonist glycine), frequency-dependent responses, desensitization, subunit composition, and more. To preserve these nuances, mechanistic approaches have been developed that employ Markov kinetic state models [6,7,8,9,10,11,12], which can represent non-linear synaptic dynamics using multiple internal states governed by first-order ordinary differential equations (ODEs). However, these computations can become increasingly burdensome as the number of modeled synapses increases. To overcome these computational burdens, synaptic models are often reduced to simple linear rise-and-decay exponential functions due to their small computational footprint, but their simplicity prevents them from replicating any form of non-linearity observed in a biological system; thus, they are incapable of accurately modeling NMDA-R dynamics.

Previously, we developed a look-up table synapse (LUTsyn) model [13] that effectively made use of the advantages of both kinetic state models (biological realism) and double exponential synapse models (fast run times). The LUTsyn model demonstrated its capability in preserving synaptic dynamics (for both AMPA-R and NMDA-R) while significantly reducing the complexity of kinetic state models by implicitly abstracting non-linearity in the form of look-up tables. In short, the look-up table holds amplitude data of the synapse’s conductance or open probability value, which are indexed by the timings of past presynaptic neurotransmitter (glutamate) pulses relative to the present presynaptic pulse. This approach allows for a fast retrieval of the synaptic response with speeds comparable to the double exponential synapse. In [13], the LUTsyn model was both trained and validated using synthetic data that was generated by a kinetic-state synapse model. The results from [13] indicate that the model was successful in speeding up a large multi-scale model of the rat hippocampus by 38-fold (when compared to simulations containing a 15-state kinetic model of NMDA-R) without sacrificing the biological realism of the kinetic synapse models.

In this study, we improve the predictive power of the NMDA-R models through optimization using experimental data. First, we trained a kinetic state model of GluN1/GluN2A NMDA-R by optimizing its rate-constant parameters in order to fit multiple experimental results. Then, we generated a new version of the NMDA-R LUTsyn model that was trained on data generated by the newly optimized kinetic state model. In this article, we first introduce the experimental NMDA-R data used for optimization, as well as the parameter-fitting process of the kinetic state model using the particle swarm optimization learning algorithm. We then describe the training protocol for the LUTsyn model and the validation of these models and discuss the implications of our results.

## 2. Materials and Methods

### 2.1. ExperimentallyConstrained Data

**Figure 1:**
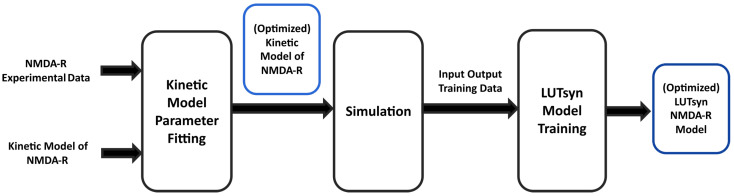
**Training pipeline of NMDA-R models |** The diagram shows the high-level procedure of using experimental data of NMDA-R dynamics to train two models of NMDA-R. First, the data is used to optimize the parameters of a kinetic model of NMDA-R to fit the experimental data. Then, the newly optimized kinetic model is used to simulate and generate input–output data to be used in training the LUTsyn model.

In the present study, experimental data of GluN1/GluN2A NMDA-R currents were used to fit and train computational models. Figure 1 illustrates the high-level steps used to train these two models. In an attempt to generate a biologically realistic synaptic model of NMDA-R, we aimed for our model to replicate both **peak amplitudes in response to varying stimulation frequency** and **the temporal profile in response to synaptic neurotransmitter release**.

To achieve the former, we used the results found in [6], in which NMDA-R-mediated currents were recorded in response to paired pulses of 1 mM glutamate (1 ms width) delivered at either 10 Hz or 100 Hz. Currents were recorded from excised patches (outside-out) of HEK293 cells recombinantly expressing GluN1/GluN2A receptors. The external medium contained 0.1 mM of glycine and no magnesium (i.e., no magnesium block) and had a pH of 8, and the membrane potential was held at approximately −100 mV. The authors developed a model that reproduced the kinetic characteristics of both native and recombinant GluN1/GluN2A currents in response to 10 Hz and 100 Hz stimulation. We use the peak open probability values predicted by this model to constrain our own model.

To achieve a model that can reproduce the synaptic temporal profile of GluN1/GluN2A NMDA-Rs, we use the results found in [14], in which the authors recorded evoked excitatory postsynaptic currents (EPSCs) from GluN1/GluN2A NMDA-Rs in wild-type CA1 pyramidal cells in the rat hippocampus. Recordings were performed using whole-cell patch-clamp mode with a holding potential of +40 mV (producing an outward NMDA-R current) and an extracellular magnesium concentration of 1 mM. The recorded EPSC trace was normalized and used to constrain the temporal dynamics of our NMDA-R model.

### 2.2. Computational and Simulation Methods

Simulations were implemented in Python 3.8 using the Tellurium library (version 2.2.2.1) [15] for kinetic models and NEURON version 8.0.0 [16] for the LUTsyn model. Kinetic models simulated in Tellurium used the variable-timestep CVODE ODE solver, while NEURON simulations of the LUTsyn model used a fixed-timestep implicit Euler solver with timesteps of 0.1 ms. Implementation of the particle swarm optimization (PSO) algorithm was performed using the Inspyred Python library [17] in parallel with AMD EPYC 7513 CPUs.

### 2.3. Kinetic Synapse Model of NMDA-R

**Figure 2:**
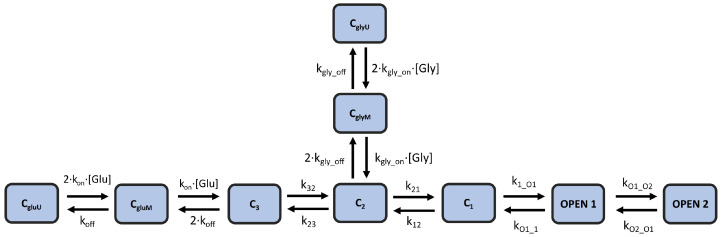
**Kinetic model of NMDA-R |** A 9-state kinetic scheme of NMDA-R that combines the models proposed in [6,20] to model the response of the receptor channel to both glutamate and glycine. The model includes 12 rate constants.

In this study, we modify and optimize an existing kinetic model of NMDA-R that originates from [6], in which the authors tested several candidate models for their ability to replicate experimental results. The best candidate was a fully sequential model that includes two states for glutamate binding, three (diliganded) closed states, and two open states. This kinetic scheme was created assuming a tonic presence of saturating glycine concentration (0.1 mM), which does not reflect the physiological conditions in the brain. The local glycine concentrations in the central nervous system are thought to be primarily regulated by glycine transporters (GlyTs), particularly the GlyT1 type at synapses, which effectively lower glycine concentrations [18,19]. Therefore, we augment this kinetic scheme by adding two glycine-binding states that come directly from [20]. Figure 2 illustrates the modified kinetic model scheme, which contains 9 total states and 12 rate constant parameters. This model (hereafter referred to as the “kinetic model”) is capable of reproducing important non-linear dynamics of the receptor, such as current potentiation, due to high-frequency stimulation, as well as the biphasic decay of NMDA-R currents in response to short pulses of glutamate.

For this kinetic model, the current or EPSC from a population of NMDA-Rs can be calculated using the total open probability (the sum of the occupancies of both open states) with Equations (1)–(4) (Table 1 identifies the used variables).

(1) $$I_{NMDAR}\left(t\right)=N\cdot \left(V-V_{rev}\right)\cdot g_{NMDAR}\left(t\right)$$

(2) $$g_{NMDAR}\left(t\right)=g_{max}\times O_{NMDAR}\left(t\right)$$

(3) $$g_{max}=\frac{g_{0}}{1+\left(\frac{Mg_{0}^{2+}}{K_{0}}\right)e^{-0.062\cdot V}}$$

(4) $$g_{0}=g_{1}\frac{g_{2}-g_{1}}{1+e^{\alpha \Psi_{m}}}$$

### 2.4. Kinetic Model Parameter Fitting

The kinetic model, using its original rate constants—taken from [6] (“low” mode) and [20] (model 2)—fit the peak amplitude responses to high-frequency square-pulse glutamate stimulation (from [6]) well but decays with a much faster time constant when stimulated with a quantal release of glutamate than the current trace reported in [14]. In order to better fit the experimental data, the kinetic model was modified using a learning algorithm to find new values of the rate constant parameters that would optimize the model’s ability to replicate the peak open probabilities due to 10 Hz and 100 Hz glutamatergic stimulation from [6], as well as reproduce the temporal dynamics in response to synaptic transmission found in experimental results of NMDA-R [14]. The particle swarm optimization algorithm (PSO) [22] was used in optimizing the parameters due to the high-dimensional parameter space (12 parameters) that must be searched. Briefly, PSO is a powerful population-based evolutionary algorithm in which multiple candidate solutions, or “particles”, distributed across the parameter space iteratively move toward the optimal (or near-optimal) solution. The algorithm aims to sample a diverse range of parameter combinations while converging toward the best solution. In each generation, each particle moves to a different position in the parameter space based on the particle’s personal best solution, its velocity, and the location of the current global best solution (among all particles). Three parameters control the weight of a particle’s personal best solution (called the **cognitive rate**), the weight of the population’s global best solution (called the **social rate**), and the weight of the particle’s velocity (called **inertia**) in determining the particle’s next position.

Optimization was performed on USC’s Center for Advanced Research Computing (CARC) nodes using AMD EPYC 7513 CPUs. PSO was implemented in Python version 3.8 using the Inspyred library [17]. Optimization consisted of 50 parallel trials in which each trial used 200 particles and was allowed to run for 14 h, resulting in 350 generations. Cognitive and social rates were set to values of 2.1, while inertia was set to 0.5. The glycine concentration was set to 0.1 mM, the $Mg^{+2}$ concentration was set to 0 mM, and the membrane potential was set at −100 mV for simulations involving repeated 1 ms square pulses of 1 mM glutamate to reflect the conditions of the experimental data coming from [6]. For simulations using quantal glutamate release as stimulation, the $Mg^{+2}$ concentration was set to 1 mM, and the membrane potential was set to +40 mV to reflect the conditions in [14]. However, the glycine concentration was not indicated in that report, so we chose a nominal physiological concentration of 0.02 mM for glycine. Synaptic transmission was achieved by simulating the dynamics of neurotransmitter release and diffusion using the equations provided in [23].

To test the fitness of a certain particle, that is, some combination of parameters, the model was simulated under three different protocols:

1 In response to three 1 ms square pulses of 1mM glutamate delivered at 10 Hz;
2 In response to three 1 ms square pulses of 1mM glutamate delivered at 100 Hz;
3 In response to a synaptic-like pulse of glutamate.

These three protocols provide multiple points of experimentally derived constraints to calibrate the kinetic model. In protocols 1 and 2, we record the three peak open probability values and compare them against the corresponding values found in [6] using the absolute percent error. The peak that corresponds to the first pulse of glutamate is identical between the 10 Hz and 100 Hz trains; therefore, one of them is discarded to prevent redundancy. In protocol 3, the synaptic current trace is normalized and compared with the normalized current trace from [14] using the normalized root mean squared error (NRMSE). More formally, optimization of the rate constants is performed by minimization of the following loss function (J(θ)).

(5) $$\;J\left(\theta \right)=\frac{1}{2}NRMSE\left(i_{syn\_norm},{\hat{i}}_{syn\_norm}^{\theta }\right)+0.1\sum_{i=1}^{3}\frac{|p_{i,10}-{\hat{p}}_{i,10}^{\theta }|}{p_{i,10}}+0.1\sum_{i=2}^{3}\frac{|p_{i,100}-{\hat{p}}_{i,100}^{\theta }|}{p_{i,100}}$$

(6) $$NRMSE\left(x_{t},y_{t}\right)=\sqrt{\frac{\sum_{t=0}^{T}{\left(x_{t}-y_{t}\right)}^{2}}{\sum_{t=0}^{T}x_{t}^{2}}}$$

θ represents the parameter set (i.e., the rate constants being tested). $i_{syn\_norm}$ represents the normalized EPSC trace taken from [14] (used as ground truth), and ${\hat{i}}_{syn\_norm}^{\theta }$ represents the normalized estimated EPSC from the model, given rate constants (θ). $p_{i,10}$ and $p_{i,100}$ represent the *i*th peak open probability value due to 10 Hz and 100 Hz stimulation from [6], respectively, while ${\hat{p}}_{i,10}^{\theta }$ and ${\hat{p}}_{i,100}^{\theta }$ are the corresponding model estimates of those peaks. *T* represents the duration of the simulation. The loss function (J(θ)) is a weighted sum of multiple losses where half the weight is put on the model’s ability to replicate the temporal profile of the synaptic case and the other half is put on reproducing frequency-dependent potentiation. At the end of PSO, the parameters are chosen to be the particle that resulted in the minimum fitness value across all 50 trials.

### 2.5. LUTsyn Model

**Figure 3:**
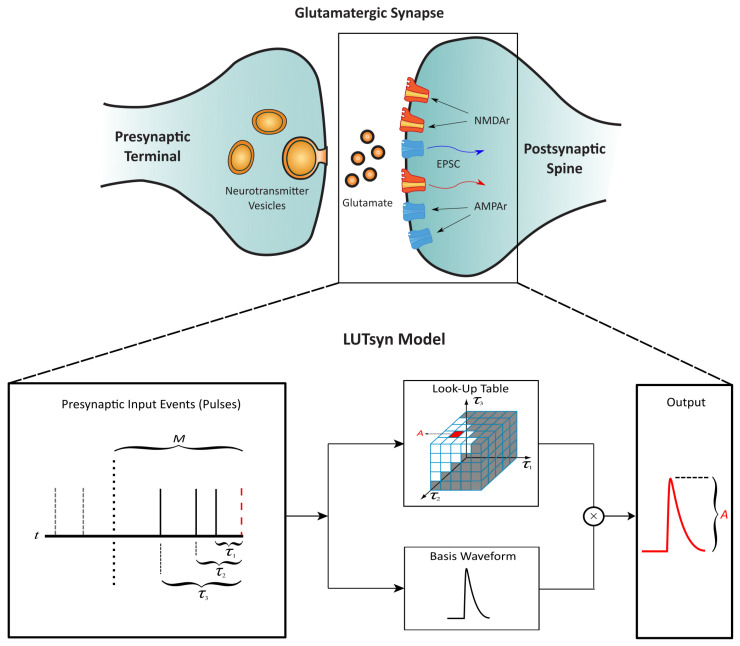
**Structure of the LUTsyn model |** The LUTsyn model is an input–output model of glutamatergic synapses containing AMPA-R and NMDA-R. The input to the model is a binary time series representing the time points at which the neurotransmitter is released by the presynaptic terminal. The input pulse time points are recorded and converted into interpulse intervals (denoted as τ) relative to the present pulse time within a memory window (*M*). The interpulse intervals are then used to access a look-up table that contains the predicted amplitude of the model’s response. The amplitude is multiplied by a basis waveform to generate the final output prediction. Figure slightly modified from [13].

The LUTsyn model is a methodology that we previously developed to abstract the input–output relationships for glutamatergic receptors AMPA-R and NMDA-R [13]. The non-linear dynamics of these receptors are assumed to be a function of the interpulse intervals of past synaptic events that occur within a certain memory window (*M*). This assumption gives rise to a look-up table data structure that stores the amplitude of a given waveform (conductance or open probability) indexed by the past interpulse intervals. The amplitude value is then multiplied by a normalized-basis waveform to generate the model’s output prediction, resulting in a computationally inexpensive model that circumvents the complexity of mechanistic approaches. Figure 3 illustrates the structure of the LUTsyn model. For NMDA-R, the basis function takes the form of a triple exponential, which is summarized in the following equations.

(7) $$O_{norm}\left(t\right)=F_{norm}\cdot \left(w\cdot e^{-t/T_{c2}}+\left(1-w\right)\cdot e^{-t/T_{c3}}-e^{-t/T_{c1}}\right)$$

(8) $$O_{NMDAR-LUT}\left(t\right)=LUT_{NMDAR}\times O_{norm}\left(t\right)$$

where $O_{norm}\left(t\right)$ represents the normalized open probability (amplitude of 1); $F_{norm}$ is a normalization factor that scales the amplitude to be 1 (based on the triplet of the used time constants); *t* represents the amount of time that has passed since the input pulse event (in ms); and $T_{c1}$, $T_{c2}$, and $T_{c3}$ are time constants (ms) that govern the dynamics and width of the waveform. The *w* parameter is a weighting factor constrained within the range of (0,1) and was optimized via grid search to have a value of 0.7384. $O_{NMDAR-LUT}\left(t\right)$ represents the open probability prediction of NMDA-R from the LUTsyn model. $LUT_{NMDAR}$ represents the amplitude value stored in the look-up table based on the values of the interpulse intervals.

The NMDA-R LUTsyn model categorizes synaptic responses by **order** (1st to 5th). For example, if three consecutive pulses occur within the memory window (*M*), the third waveform (i.e., the response to the third pulse) would be categorized as a third-order response. Synaptic events beyond the 5th order are treated as 5th-order responses by the model such that only the five most recent pulses are considered in the prediction of the output. If the model has no synaptic inputs after *M* time has passed, then the next pulse is treated as of the 1st order. The LUTsyn model uses a different triplet of time constants for each order. The process of finding the time constants for each order are described in the LUTsyn Model Training section.

### 2.6. LUTsyn Model Training

The newly optimized kinetic model was used to generate continuous open probability time-series data in response to random pulse trains representing synaptic events (i.e., glutamate release) using the Tellurium simulation framework [15]. First, the concentration value of glycine was set to 0.02 mM, and the $Mg^{+2}$ concentration was set to 1 mM for the kinetic model to reflect more physiological values found at glutamatergic synapses. Second, an intermediate mechanism that models glutamate diffusion was implemented to convert the input binary signal of pulse times to a continuous glutamate concentration input signal [23] for the kinetic model. This data was then used to train and validate the NMDA-R LUTsyn model using the same process described in detail in [13]. First, the time constants of each pulse order were estimated by the following process:

1 Generate a Poisson pulse train of synaptic events.
2 Generate the NMDA-R response to the pulse train using the kinetic model.
3 Categorize each response’s waveform as 1st–5th order.
4 Calculate the mean response of each order.
5 Estimate the time constants of a triple exponential that best fit the mean response of each order using PSO.

The loss function for optimizing the time constants via PSO is the sum of the NRMSE between the two responses and the absolute percent error between both responses’ full duration at half maximum (FDHM). Optimization of time constants was performed using 50 particles, 200 generations, inertia = 0.5, cognitive rate = 2.1, and social rate = 2.1. The input Poisson pulse train had a mean frequency of 4 Hz and lasted 40 s.

Second, the look-up table containing the peak open probability values that depend on the timing of past synaptic events was generated by simulating all possible firing patterns with the kinetic model (within a certain time memory window of *M* = 1000 ms and a granularity of δ = 5 ms) and storing the amplitude of the waveform response in the data structure described in full detail in [13].

## 3. Results

### 3.1. Validation of Kinetic Model

**Figure 4:**
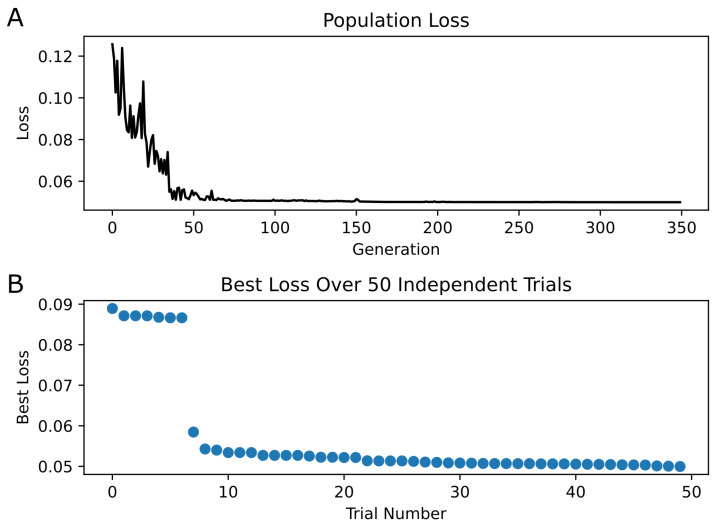
**PSO Results |** (**A**) The best loss in each generation is plotted for only the best trial (i.e., the trial containing the individual with the minimum loss across all trials). Convergence of loss appears at approximately generation 70. (**B**) The best loss of each of the 50 trials is shown, sorted in descending order.

**Figure 5:**
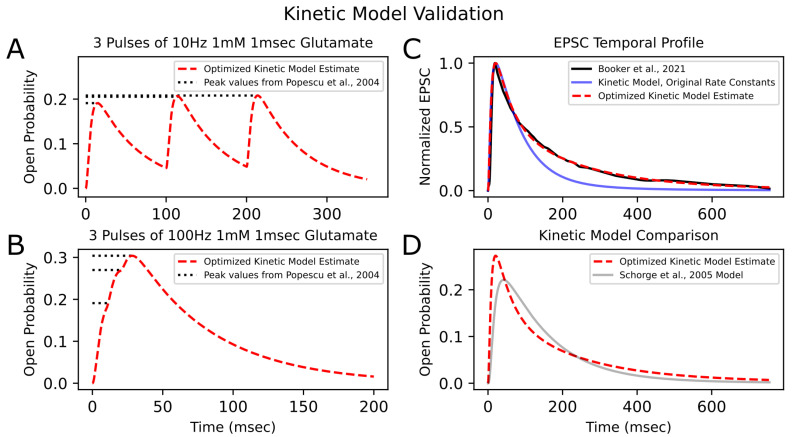
**Kinetic model validation |** After optimizing the rate constants of the kinetic model to fit experimental results, the model’s ability to reproduce peak open probability values from (Popescu et al., 2004) [6] from 10 (**A**) and 100 Hz (**B**) stimulation is shown. Dashed red traces are the model’s estimate. Dotted horizontal lines represent peak open probability values from [6]. (**C**) The model’s normalized response to synaptic release of glutamate (dashed red line) is superimposed with the experimental EPSC trace (solid black line) from (Booker et al., 2021) [14] (digitized, cubic-spline interpolated, and normalized). The kinetic model’s response before rate-constant optimization is also shown (solid blue line) and has a faster decay phase than the experimental EPSC trace. (**D**) The open probability trace of the optimized kinetic model is compared to that of another NMDA-R GluN1/GluN2A Markovian model (gray line) from (Schorge et al., 2005) [10], both in response to a synaptic release of glutamate.

Optimization of the kinetic model was performed using PSO with 200 particles per trial over 50 trials. The minimum loss value for the kinetic model across all trials was 0.05. Figure 4 shows the convergence of the loss function over the generations, as well as the final best loss of each trial. Figure 5 illustrates the newly optimized kinetic model’s ability to achieve its three objectives. In Figure 5A,B, the kinetic model is shown to precisely replicate the peak open probability values presented in [6] at both 10 Hz and 100 Hz glutamate stimulation. In Figure 5C, the EPSC temporal profile from [14] is superimposed with estimate traces of the kinetic model using both its original rate constants and optimized rate constants. The optimized kinetic model accurately replicates the dynamics of the experimental trace, while the original kinetic model was unable to reproduce the slower decay phase of the synaptic response. The weighted decaying time constants ($\tau_{w}$) were estimated for these three traces by fitting a biexponential to the decay phase of each response. The $\tau_{w}$ values for the experimental response from (Booker et al., 2021) [14] and the optimized kinetic model were very similar, with values of 148.48 ms and 147.43 ms, respectively, while the kinetic model with original rate constants had a significantly faster $\tau_{w}$ of 85.86 ms.

For further validation of the optimized kinetic model, open probability traces in response to synaptic glutamate release were compared to that of an existing 15-state Markovian model of NMDA-R GluN1/GluN2A from (Schorge et al., 2005) [10] (hereafter referred to as the “Schorge model”; Figure 5D). The two models produce relatively similar first-order responses, but the optimized kinetic model had a faster rise time and faster initial decay phase and reached a higher open probability peak than the Schorge model. The decay phase of the Schorge model’s response approached a monoexponential with a single time constant of 149.93 ms, similar to the weighted time constant of the optimized kinetic model, which was 147.43 ms. The optimized kinetic model had an faster initial decay than the Schorge model but then had a later, slower decay phase, which can be explained by its biexponential dynamics, with the two time constants being 63.76 ms and 257.61 ms. Differences between the two models’ responses may be attributed to differences in their respective studies’ methods. For example, (Popescu et al., 2004) [6] studied receptors expressed in HEK293 cells, whereas (Schorge et al., 2005) [10] studied receptors expressed in *Xenopus* oocytes.

**Figure 6:**
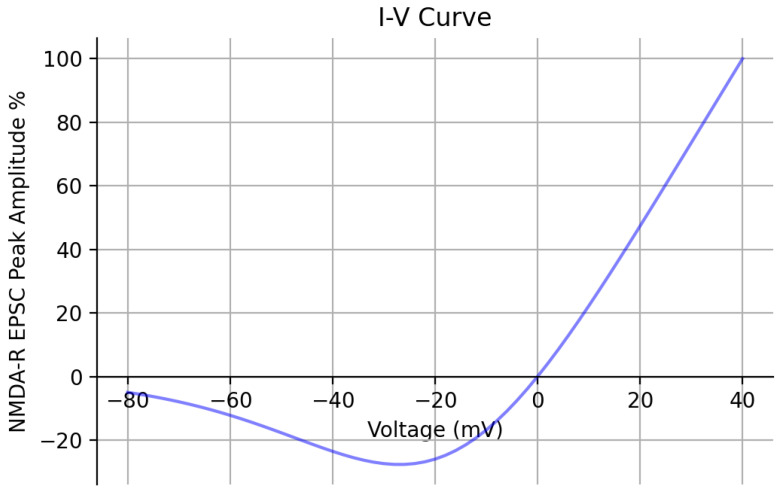
**Kinetic model I-V curve |** The current–voltage relationship of the optimized kinetic model is shown across a range of holding potentials spanning from −80 mV to +40 mV. The peak amplitude of the EPSCs are recorded and reported as a percent with respect to the peak value recorded at +40 mV. The $Mg^{+2}$ concentration was set to 1 mM.

Lastly, additional validation of the optimized kinetic model was performed by obtaining its I-V relationship (Figure 6). Peak EPSC amplitude values were recorded, with the $Mg^{+2}$ concentration set to 1 mM at each holding potential and reported as a percent with respect to the peak value recorded at +40 mV. The I-V curve has a comparable shape to the experimentally obtained I-V curve from [24]. Both I-V curves exhibit a characteristic region of negative slope conductance caused by the $Mg^{+2}$ blockade. Table 2 reports the values of the original and newly optimized rate constants of both kinetic models as a result of PSO.

### 3.2. Validation of LUTsyn Model

**Figure 7:**
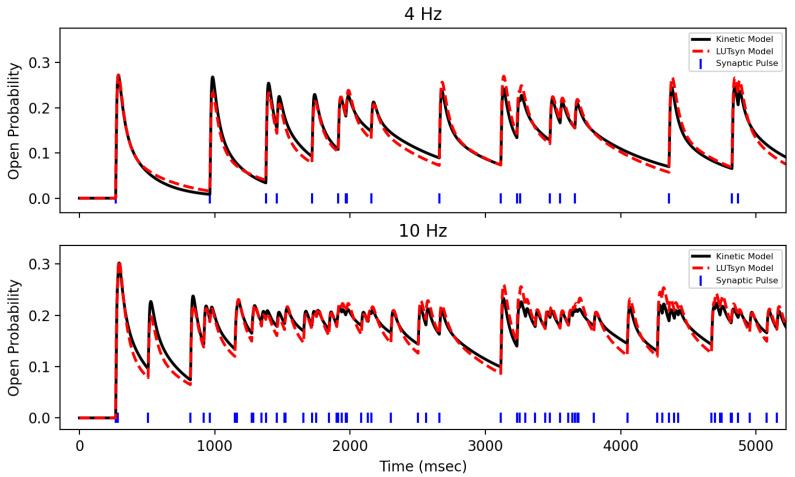
**Validation of NMDA-R LUTsyn Model |** The LUTsyn model’s prediction of NMDA-R normalized open probability (dashed red) is compared with the same response from the optimized kinetic model (black) over 4 Hz (**top**) and 10 Hz (**bottom**).

After training the LUTsyn model on data generated by the optimized kinetic model, the LUTsyn model was validated against two novel datasets. Validation datasets were 20 s long open probability traces in response to Poisson trains with mean frequencies of 4 and 10 Hz generated by the optimized kinetic model. Validation was performed by measuring the NRMSE between the LUTsyn model’s predicted open probability responses to these validation sets. NRMSEs of 0.115 and 0.078 were achieved for 4 Hz and 10 Hz, respectively. Figure 7 shows a comparison of LUTsyn’s prediction with the kinetic model’s data.

## 4. Discussion

In the present study, we described the methodology we applied to constrain two computational models of GluN1/GluN2A NMDA-R with respect to experimental results. These results contained multiple data points, which allowed us to constrain both the amplitude and temporal profile of our models. The particle swarm optimization (PSO) algorithm was utilized to optimize the rate constants of a kinetic model that, in its original state, could not replicate the temporal dynamics of the experimental synaptic data using its original rate constants. The optimization process relied on three experimental constraints: fitting of peak amplitude responses to 10 Hz stimulation, fitting of peak amplitudes to 100 Hz stimulation, and fitting of the temporal profile in response to synaptic glutamate release. Once the kinetic model was optimized, we validated it by comparing its response to that of another Markov kinetic state model and found relatively similar responses between the two models but with differences in decaying time constants and peak open probability values. Moreover, the I-V curve for the kinetic model was generated, showing the characteristic negative slope conductance region in the negative membrane potential range that was shown in an experimentally found I-V curve of NMDA receptors [24], further validating our model.

The kinetic model was then used to generate a rich dataset that was used to train the LUTsyn model—a computationally efficient input–output model that aims to closely mimic the response of the optimized kinetic model with a minimal computational footprint. The results obtained with the optimized kinetic model demonstrated its ability to faithfully reproduce the response of the biological system in terms of both peak amplitude response to high-frequency stimulation and the temporal dynamics in response to a synaptic pulse of glutamate. Subsequently, we showed the LUTsyn model’s ability to replicate the response of its kinetic counterpart, especially in terms of its non-linear dynamics, while using a minimal computational and memory footprint. Notably, all quantitative details highlighting the superior computational efficiency of the LUTsyn model were described in large-scale network-level simulations in a previous study (Pham et al., 2021) [13]. The results demonstrated a clear advantage of the LUTsyn model, which offered a 38-fold increase in computational speed during large-scale simulations of a rat hippocampal model compared to simulations that used a 15-state kinetic model for NMDA-R. As the structure of the LUTsyn model is preserved, these results can be extended to the model presented here. Finally, while the LUTsyn model footprint is comparable to the often used double or triple exponential linear models, its ability to faithfully replicate the non-linearities of the biological system makes it a powerful alternative that offers a balance of biological realism and high speed.

The primary goal underlying the development of the LUTsyn model was to create a biologically realistic model using multiple experimentally derived constraints capable of accurately replicating the complex non-linear behavior of the receptor with a small computational footprint so that it could be used in a large-scale neuronal network model. Consistent with this original objective, an updated kinetic NMDA-R model was developed, and the NMDA-R LUTsyn model was successfully updated to closely mimic dynamics recorded in experimental data.

Given the critical role that NMDA receptors have been established to play not only in synaptic transmission but also in long-term potentiation and synaptic plasticity in general, accurately modeling their dynamical response constitutes a priority. We have demonstrated that the models presented here achieve this critical goal and that the LUTsyn model provides an excellent option for integration in large-scale models. Both NMDA-R models presented here and their respective code will be made available to the community via an online repository (GitHub) to enable future computational studies that incorporate lightweight and highly realistic receptor dynamics.

## Figures and Tables

**Table 1:** Variables used in calculating NMDA-R EPSC.

Variable Name	Description	Notes
$O_{NMDAR}\left(t\right)$	Open probability of NMDA-R at time *t*	-
$I_{NMDAR}\left(t\right)$	EPSC mediated by a population of NMDA-Rs at time *t*	Derived from Ohm’s Law
*N*	Number of NMDA-Rs present	-
*V*	Membrane potential	Units of mV
$V_{rev}$	Reversal potential of NMDA-R	Set to 0 mV
$g_{NMDAR}\left(t\right)$	Expected value of single-channel conductance of NMDA-R at time *t*	Units of pS
$g_{max}$	Single-channel conductance of NMDA-R when factoring the voltage-dependent magnesium block	Equation (3) was derived from [21] (units of pS)
$g_{0}$	Single-channel conductance of NMDA-R when magnesium is absent	Equation (4) was derived from [12] (units of pS)
$g_{1}$	Open-state conductance with 1 glutamate molecule bound	From [12] (units of pS)
$g_{2}$	Open-state conductance with 2 glutamate molecules bound	From [12] (units of pS)
$Mg_{0}^{2+}$	External magnesium concentration	Units of mM
$K_{0}$	Equilibrium constant of magnesium	Value set to 3.57 from [21]
α	Steepness of voltage-dependent transition from $g_{1}$ to $g_{2}$	from [12]
$\Psi_{m}$	Holding potential	Units of mV

**Table 2:** Rate constants of the kinetic model before and after PSO.

Rate Constant (Units)	Original Value	New Value
$k_{on}$ (μM^−1^ s^−1^)	17	46
$k_{off}$ (s^−1^)	60	86
$k_{32}$ (s^−1^)	127	208
$k_{23}$ (s^−1^)	161	152
$k_{21}$ (s^−1^)	580	1200
$k_{12}$ (s^−1^)	2610	5000
$k_{1\_O1}$ (s^−1^)	2508	1130
$k_{O1\_1}$ (s^−1^)	2167	4823
$k_{O1\_O2}$ (s^−1^)	3449	6998
$k_{O2\_O1}$ (s^−1^)	662	278
$k_{gly\_on}$ (μM^−1^ s^−1^)	5	1
$k_{gly\_off}$ (s^−1^)	12	24

## Data Availability

Example code that runs the trained kinetic model and LUTsyn model and compares their open probability traces is available. Code is available in our public Github repository, including instructions on how to execute it: https://github.com/duytanph/exp_constrained_NMDAR_models (accessed on 23 June 2025).

## Abbreviations

The following abbreviations are used in this manuscript:

NMDA-R	N-methyl-D-aspartate Receptor
LUTsyn	Look-Up Table Synapse
PSO	Particle Swarm Optimization
NRMSE	Normalized Root Mean Squared Error
EPSC	Excitatory Postsynaptic Current
